# An Update on DOTA-Peptides PET Imaging and Potential Advancements of Radioligand Therapy in Intracranial Meningiomas

**DOI:** 10.3390/life15040617

**Published:** 2025-04-07

**Authors:** Viviana Benfante, Ignazio Gaspare Vetrano, Muhammad Ali, Pierpaolo Purpura, Cesare Gagliardo, Paola Feraco, Costanza Longo, Tommaso Vincenzo Bartolotta, Patrizia Toia, Oriana Calisto, Albert Comelli, Massimo Midiri, Pierpaolo Alongi

**Affiliations:** 1 Advanced Diagnostic Imaging-INNOVA Project, Department of Radiological Sciences, A.R.N.A.S. Civico Di Cristina e Benfratelli Hospitals, P.zza N. Leotta 4, 90127 Palermo, Italy; pierpaolopurpura@gmail.com (P.P.); pierpaolo.alongi@unipa.it (P.A.); 2 Department of Neurosurgery, Fondazione IRCCS Istituto Neurologico Carlo Besta, 20133 Milan, Italy; ignazio.vetrano@istituto-besta.it; 3 Department of Biomedical Sciences for Health, University of Milan, 20133 Milano, Italy; 4 Ri.MED Foundation, Via Bandiera 11, 90133 Palermo, Italy; amuhammad@fondazionerimed.com (M.A.); acomelli@fondazionerimed.com (A.C.); 5 Department of Biomedicine, Neuroscience and Advanced Diagnostics (BiND), University of Palermo, 90127 Palermo, Italy; cesare.gagliardo@unipa.it (C.G.); tommasovincenzo.bartolotta@unipa.it (T.V.B.); massimo.midiri@unipa.it (M.M.); 6 Neuroradiology Unit, University-Hospital Paolo Giaccone, 90127 Palermo, Italy; 7 Centre for Medical Sciences (CISMed), University of Trento, 38122 Trento, Italy; 8 Nuclear Medicine Unit, A.R.N.A.S. Civico Di Cristina e Benfratelli Hospitals, P.zza N. Leotta 4, 90127 Palermo, Italy; costanza.longo@arnascivico.it; 9 Department of Radiology, AOUP Paolo Giaccone, Via del Vespro 129, 90127 Palermo, Italy; 10 Nuclear Medicine Unit, Department of Biomedical and Dental Sciences and Morpho-Functional Imaging, University of Messina, 98122 Messina, Italy; oriana.calisto@gmail.com

**Keywords:** PET, meningioma, DOTATOC, DOTANOC, DOTATATE, radioligand therapy

## Abstract

Meningiomas arise from the meningeal layers covering the central nervous system structures. Although most are benign, meningiomas can still cause neurological morbidity due to the mass effect and compression of the surrounding parenchyma. The prognosis also depends on several factors such as growth pattern or location. Morphological imaging approaches, such as MRI and CT, that emphasize intracranial calcifications, vascular patterns, or invasion of major vessels act as the basis of the diagnosis; PET/CT imaging is a valuable diagnostic tool for assessing somatostatin receptor activity in tumors. It enables the visualization and quantification of somatostatin receptor expression, providing insights into tumor biology, receptor status, and potential therapeutic targets. Aside from radiosurgery and neurosurgical intervention, peptide receptor radionuclide therapy (PRRT) has also shown promising results. Somatostatin receptors 1 and 2 are nearly universally expressed in meningioma tissue. This characteristic is increasingly exploited to identify patients eligible for adjuvant therapy using DOTA-conjugated somatostatin receptor-targeting peptides PET. In the treatment of relapsed/refractory meningiomas, PRRT is increasingly considered a safe and effective therapeutic option. It is often supported by artificial intelligence strategies for dose optimization and side-effect monitoring. The objective of this study is to evaluate the safety and benefits of these strategies based on the latest findings.

## 1. Introduction

Meningiomas present a significant clinical and radiological diagnostic challenge [1], primarily due to the high prevalence of cases categorized as “rarely localized” meningiomas. Moreover, morphologically [2] and functionally [3], meningiomas exhibit similarities to arachnoid cap cells. The presence of meningiomas has been shown to correlate with a marked increase in arachnoid villi within the major venous sinuses [4]. This pathology remains a focal point of ongoing research, particularly concerning the potential application of various diagnostic and therapeutic strategies.

**Figure 1:**
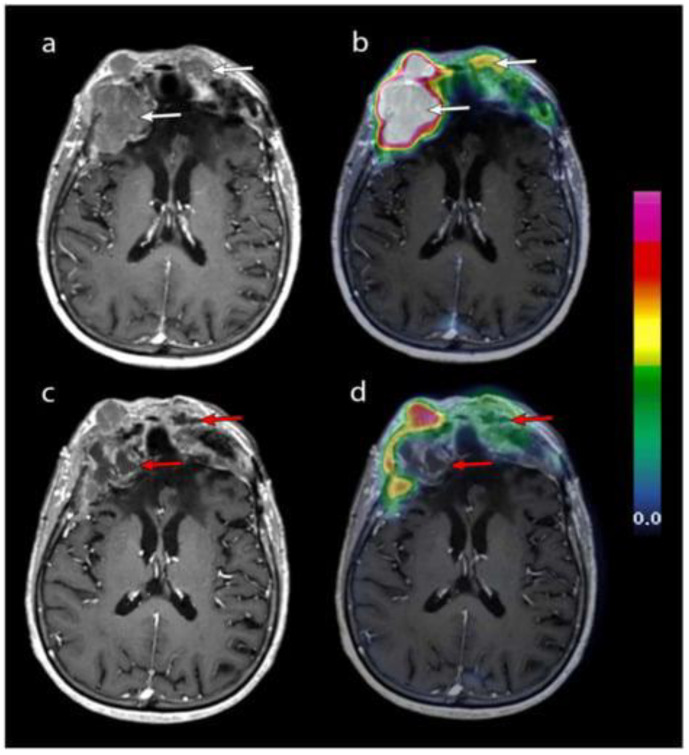
Contrast-enhanced T1-weighted axial MRI after the second (**a**) and fourth (**c**) cycles of PRRT, merged with 68Ga-DOTATOC PET (panels (**b**) and (**d**), respectively). Two frontal lesions (white arrows) exhibiting high 68Ga-DOTATOC uptake underwent necrosis after four cycles (red arrows). However, the underlying cause of this necrosis remains uncertain, as it could result from a direct effect of PRRT or natural tumor progression. Reprinted from [8] under CC BY license.

**Figure 2:**
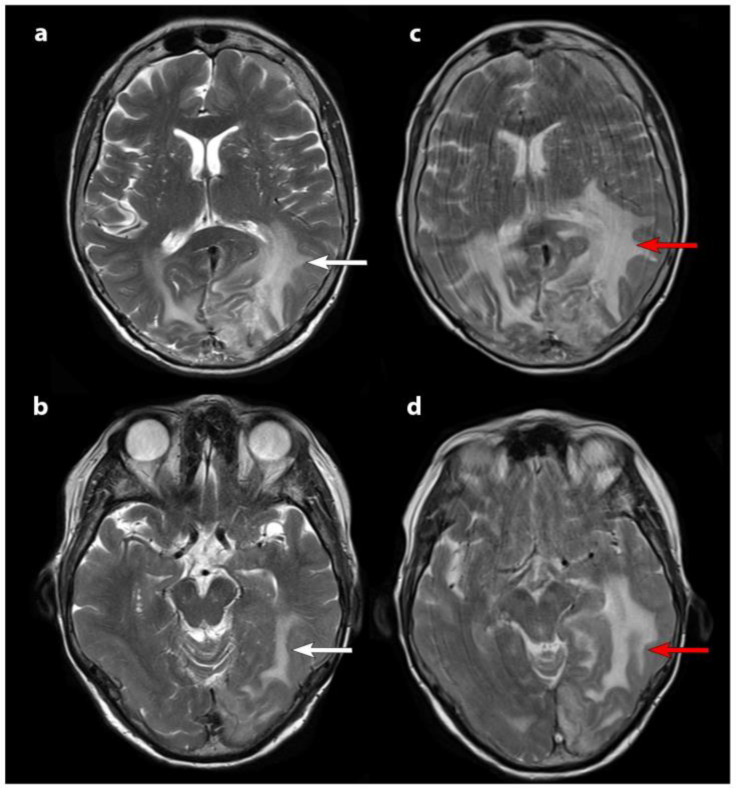
T2-weighted axial MRI scans: pre-treatment MRI images (panels (**a**,**b**)) and post-second cycle PRRT scans (panels (**c**,**d**)). White arrows indicate baseline brain edema, which worsens after the second cycle, as shown by red arrows. The increased motion artifacts in the second MRI (panels (**c**,**d**)) correspond to the patient’s clinical decline, aligning with radiological progression. Reprinted from [8] under CC BY license.

In recent years, nuclear medicine has increasingly focused on the development of theranostic markers [5,6], which are tracers designed to target overexpressed molecules in a tumor. These markers utilize diagnostic radionuclides for positron emission tomography (PET) imaging, along with α- or β-emitting radionuclides for therapeutic purposes [7]. Theranostic applications in meningiomas involve radiolabeled somatostatin receptor (SSTR) ligands, as type 2 SSTR receptors are known to be overexpressed in these neoplasms. Figure 1 shows an imaging acquisition of a woman undergoing contrast-enhanced axial T1-weighted MRI coupled with 68GA-DOTATOC PET, after several peptide receptor radiopharmaceutical therapy (PRRT) cycles [8]. The patient selection and monitoring in PRRT for meningiomas is crucial for early detection of possible therapy failure. To further illustrate the spectrum of responses to PRRT, a case of treatment failure in a patient with WHO grade II multiple frontal meningiomas has been reported. Clinically, the most common adverse event observed in this patient was asthenia, while two patients experienced transient seizures with increased frequency. Notably, increased perilesional edema on MRI was not a common finding among the treated patients in this study. However, this particular patient had substantial pre-existing brain edema at baseline, which worsened despite escalated doses of corticosteroids, ultimately proving refractory to treatment. The annotated figure highlights these radiological changes, supporting the importance of PET in patients treated with PRRT for meningiomas (Figure 2).

Although not part of standard diagnostic management, PET and single-photon emission computed tomography (SPECT) imaging can aid in detecting challenging meningiomas such as skull base tumors or optic nerve sheath meningiomas [9]. PET imaging may be accurate in the molecular characterization of the lesion [10], and in the case of bone involvement [11], nuclear medicine techniques offer significant contributions. All these features make SSTR PET particularly useful, even if not specific, both for the accurate differentiation between post-therapeutic scar tissue and tumor recurrence [12] and for treatment planning, for example, for the definition of target volumes before radiotherapy [13].

In the diagnostic settings, meningiomas are evaluated on PET using SSTR ligands labeled with [68Ga]Gallium DOTA-Tyr3-octreotate (DOTATATE), DOTA-Tyr3-octreotide (DOTATOC), and DOTA-1-Nal(3)-octreotide (DOTANOC). Recently encouraging results have been found using [18F]Fluorine-labeled [18F]SiTATE, a [Tyr3]-octreotate conjugated with SiFAlin, as well as [64Cu]Copper-labeled DOTATATE [12,14,15]. The clinical usefulness of SPECT with [111In]In-pentetreotide or [99mTc]Tc-EDDA-TRYCINE-HYNIC-TOC is marginal [16].

**Figure 3:**
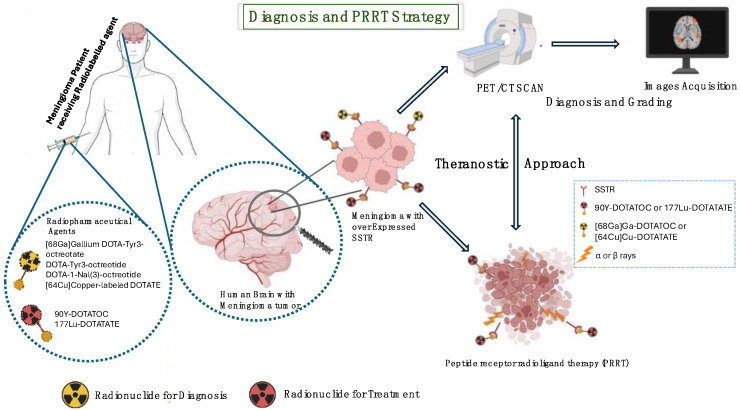
Meningioma diagnostic approaches and PRRT therapy overview.

In the theranostic field, PRRT represents a new treatment option based on targeted β-emitters radionuclides directed to peptides, such as SSTRs [17], which selectively irradiate targeted tumor lesions by later depositing their high energy within a short range in tissues. Among the most commonly used radionuclides in radiopharmaceutical therapy are Yttrium 90 [90Y] and Lutetium 177 [177Lu]. Beta decay makes them both interesting for therapeutic properties. In detail, [90Y] has a half-life of 64.1 h, maximum energy of the emitted β particles equal to 2284 keV, and maximum range of the produced β particles equal to 11.3 mm; [177Lu] has a half-life of 6.7 days, maximum energy of emitted β particles of 497 keV, and a maximum range of emitted β particles of 1.8 mm. In randomized trials, PRRT is developed for the treatment of refractory meningiomas [12] in patients with recurrent meningiomas after surgery and radiotherapy. Toxicity after PRRT is investigated to define safe and effective protocols. PET staging and radiometabolic therapy are intended to reduce side effects and reduce the risk of disease progression or relapse after surgery, thus reducing therapeutic toxicity and recurrence and progression rates. The purpose of this paper is to review the most recent results for both diagnostic PET and radioligand therapy for meningiomas. An overview of current nuclear medicine diagnostic and therapy approaches in meningiomas is displayed in Figure 3.

## 2. Materials and Methods

Comprehensive literature searches were performed in November 2024 using Pubmed, EMBASE, and Scopus. The search strings included the keywords “meningiomas”; “PET”; “DOTA-peptides”; “PRRT”; “nuclear medicine”; and any derivative of the aforementioned terms. Publications before 2022, studies that did not describe PET and radioligand therapy in meningiomas, and publications in a language other than English were excluded. Articles were checked for relevance using the title and abstract; then, full-text articles were obtained and reviewed. Given the nature of this review and the relative nonconventional use of nuclear medicine in this field, the authors selected the papers, including case series, based on potential values of research/clinical applications in meningiomas.

## 3. Clinical Evidence in PET Imaging and Radioligands for Meningiomas

Currently, ongoing studies on [68Ga]Ga-DOTATOC, [68Ga]Ga-DOTATATE, [64Cu]Cu-DOTATATE, [64Cu]Cu-DOTATOC, [68 Ga]Ga-DOTANOC, and [18F]SiTATE can detect meningioma tissue with high sensitivity and specificity, as SSTR-directed PET imaging can be useful for multiple diagnosis, the mapping of meningioma size, recognition of bone infiltration, and differentiating between post-therapeutic scar tissue and cancer spread [12].

### 3.1. Diagnostic Approach to Meningioma Through PET Imaging

The management of meningiomas involves the monitoring of lesion progression and severity, as these parameters are closely associated with the likelihood of recurrence or malignant transformation. In most cases, meningiomas are benign, presenting with either stable sizes or slow growth. The rate of tumor growth plays a crucial role in determining the appropriate timing for intervention or treatment. A single report demonstrated optimal performances with a high staging sensitivity and a dramatic affinity change by using 68Ga-DOTATOC and 18F-FDG in estimating the progression from atypical meningioma grade II to anaplastic meningioma grade III [18]. Differential diagnosis between metastases with meningeal implant and meningiomas represents crucial diagnostic information; 68Ga-DOTATOC PET/CT has been demonstrated to be a valuable tool in this field [19].

The use of semiquantitative parameters (e.g., SUV values) can be a valuable approach to helping a nuclear medicine reporter in the characterization of meningiomas by using PET imaging. In a retrospective monocentric study, the evaluation of semiquantitative parameters in 68Ga-DOTANOC PET/CT with intracranial meningiomas has been performed in thirty-two patients with grade I meningiomas, five patients with grade II meningiomas, and two patients with anaplastic (grade III) meningiomas demonstrating an increase in median SUVmax values related to the WHO grade [20]. Due to the clear delineation between the tumor border and the background in all cases, it was possible to determine how far the meningioma had spread intracranially.

As for the diagnostic differentiation of lesion grade in disease, semiquantitative parameters of PET may help the clinician in other settings. Firstly, one of the main important issues is therapy assessment. Indeed, the utility of [68Ga]-DOTATATE PET/MRI in assessing meningioma response 6–12 months after radiosurgery [21] was evaluated in a prospective study with 27 meningioma patients. Gadolinium-enhanced PET/MRI was performed for follow-up. The following parameters were used for evaluation: maximum absolute value of standardized uptake (SUV); SUV ratio (SUVRSSS), which refers to the blood pool of the superior sagittal sinus (SSS). The association between SUVRSSS change magnitude and progression-free survival (PFS) was calculated using Cox regression, with post-irradiation SUV and SUVRSSS decreasing by 37.4% and 44.4%, respectively, and a hazard ratio of 0.48 per 10-unit reduction in SUVRSSS in the SRS cohort.

Molecular imaging is also crucial in clinical monitoring and guiding patient care, as shown in a case of chordoid meningioma [22]. PET imaging using 68 Ga-DOTATATE and 18 F-FDG during post-surgical follow-up showed local recurrences and extracranial metastases. Although this is a case report, it highlights the significant impact of molecular imaging on identifying invasive and malignant meningiomas. In a prospective study [23] to evaluate the molecular profile of patients with grade III meningioma, patients were scanned by PET/MR imaging with gallium 68 (68Ga) DOTATATE. Patients were stratified by de novo versus secondary progressive status, and differences in standard PET uptake, molecular profiles, and clinical outcomes were evaluated. Patients with multiple surgeries had significantly lower progression-free survival than de novo patients. Moreover, secondary progressive tumors had more mutations and higher gallium 68 (68Ga) DOTATATE PET uptake.

In the evaluation of the impact of therapy work-up, Perlow et al. [24] demonstrated the efficiency and benefit that patients with intracranial meningiomas can derive from treatment guided by 68Ga-DOTATATE positron emission tomography (PET). In total, 60 patients were included in this paper, where most patients were diagnosed with grade I and II meningiomas, with a similar percentage of each, and 5% of cases included patients with grade III meningiomas. Five patients did not show any meningioma grade. In this study based on PET results, the choice of radiation therapy was the main treatment option for most patients. However, just a small group underwent follow-up, and one patient underwent multiple surgeries. It is important to note that one patient with grade III meningioma developed toxicity after PET-guided radiation treatment.

The recent development of new artificial intelligence has been integrated into a research contest in the evaluation of the predictive value of imaging features. Sarah et al. [25] conducted a retrospective study to analyze the relationship between somatostatin receptor subtypes (SSTR 1–5) and SUVmax in meningioma patients, as measured by Gallium-68 DOTA-D-Phe1-Tyr3-octreotide PET ([68Ga]Ga-DOTATOC PET). Additionally, a radiomic model based on apparent diffusion coefficient (ADC) maps from diffusion-weighted MRI was developed to predict SUVmax. The study included 51 patients who underwent both MRI and [68Ga]Ga-DOTATOC PET before surgery. A random forest regression model was used to assess SUVmax, and its validity was evaluated via nested cross-validation. SSTR subtype distribution was quantified in 18 surgical samples and compared to SUVmax values. The model showed a strong correlation with SUVmax in all 100 repeats (mean Pearson correlation coefficient r = 0.42), and SSTR subtypes 2A, 2B, and 5 were significantly correlated with SUVmax. The radiomic model based on ADC maps effectively predicted SUVmax and demonstrated the utility of combining imaging techniques for improved assessment in meningioma patients.

In patients undergoing radiotherapy, the correlation and complementary integration of diagnostic imaging can be a crucial component of the treatment plan. In this setting, the efficacy of adding 68Ga-DOTATATE PET/MRI to standard MRI for precise volume estimation in Gamma Knife stereotactic radiosurgery (GKSRS) for meningiomas was evaluated in a prospective study [26]. Seventeen WHO grade I meningioma patients underwent pre-treatment PET/MRI in addition to standard MRI. Five observers independently delineated the gross tumor volume (GTV) based on MRI alone (GTVMRI) and with PET/MRI (GTVPET/MRI), and interobserver concordance was evaluated using Cohen’s kappa statistic (CKS), Dice similarity coefficient (DC), and Hausdorff distance (HD). Results obtained by PET/MRI optimized the time required to delineate palpable tumor volume. There was a statistically significant correlation between inter-rater and intra-rater reliability and model performance. PET/MRI did not affect contour reproducibility. These findings suggest that PET/MRI enhances target volume delineation in GKSRS for meningiomas but may introduce discrepancies among clinicians.

Recently, the utility of 68Ga-DOTATATE PET/CT in differentiating neuroaxis tumors in the head and neck [27], including meningiomas, was tested in a retrospective study. In total, 70 neuroaxis lesions from 52 patients were analyzed, with 31% pathologic confirmation. Lesions were classified based on pathology and radiologic diagnosis. Statistical analysis of 68Ga-DOTATATE uptake (median SUVmax 62; IQR 89) showed that paragangliomas had significantly higher levels than non-paragangliomas. Schwannomas exhibited minimal uptake, while meningiomas and other neuroaxis lesions showed intermediate levels. Receiver operator characteristic analysis yielded an area under the curve of 0.87 for distinguishing paragangliomas from other lesions. It also achieved 0.97 for schwannomas versus all others. The study concluded that marked 68Ga-DOTATATE uptake (>50 SUVmax) strongly supports a diagnosis of paraganglioma, while low to moderate uptake excludes schwannomas but is nonspecific for other tumors, including meningiomas.

Because of the potential aggressiveness of several meningiomas, prognostic stratification is considered clinically relevant in clinical practice. Teske et al. evaluated the prognostic impact of postoperative [68Ga]Ga-DOTA-TATE PET/CT for progression-free survival (PFS) estimation in resected WHO grade I meningiomas [28]. In total, 46 patients with 49 tumors underwent postoperative MRI and PET/CT, followed by regular MRI surveillance for tumor progression. During a median follow-up of 52 months, local tumor progression occurred in 14% (7/49) of cases. Increased [68Ga]Ga-DOTA-TATE PET uptake was significantly associated with increased progression risk and shorter PFS, while MRI findings were not predictive. Negative PET findings in 20 patients were associated with recurrence-free status. On MRI, recurrences were consistently adjacent to areas identified as tumor remnants on PET, and gross tumor volumes were higher on PET than on MRI. These results highlight the sensitivity of [68Ga]Ga-DOTA-TATE PET/CT in detecting tumor remnants, improving PFS prediction, and aiding in adjuvant radiotherapy planning for resected meningiomas.

The complementary role of PET and MRI is also crucial in the assessment of local diffusion of disease. A study compared somatostatin receptor PET/MRI with contrast-enhanced MRI alone for detecting and localizing head and neck neuroendocrine tumors (NETs) and assessed the utility of vertex-to-thigh imaging [29]. In this study of about 30 patients with NETs, such as meningiomas and paragangliomas, PET/CT imaging was used along with contrast-enhanced MRI, with the PET/CT being fused with the MRI to allow for a better analysis. Among 25 patients with somatostatin receptor-positive lesions, PET/MRI and MRI findings were comparable in 11 cases, while PET/MRI revealed more extensive disease in seven patients and additional lesions in nine patients. Vertex-to-thigh imaging showed metastatic disease in 1 of 17 patients, with incidental findings in eight. Compared to MRI alone, somatostatin receptor-PET is demonstrated to detect additional lesions and more extensive disease due to its superior spatial resolution enhancing anatomic delineation over MRI alone. NETs of the head and neck can be better diagnosed and treated with this planning method.

Rodriguez and coworkers demonstrated the feasibility of integrating 68 Ga-DOTATATE PET/MR imaging into after-treatment radiation therapy strategies for patients with moderate-risk meningiomas [30]. When a decision problem involves continuous risk over time, when critical events can occur more frequently than once, and when time is a critical parameter, it is necessary to use analysis models that can assess healthcare cost-effectiveness on disease progression. The Markov model is one of the most widely used models in clinical settings based on information about the health status and history of patient cohorts [31]. Using management recommendations and institutional data, the analysis incorporated Markov models to estimate quality-adjusted life-years (QALYs) and performed cost-effectiveness analyses with societal affordability thresholds of USD 50,000/QALY and USD 100,000/QALY. Results demonstrated that 68 Ga-DOTATATE PET/MR imaging provided higher QALYs (5.47 vs. 5.05) at a slightly increased cost (USD 404,260 vs. USD 395,535) compared to MRI alone. The incremental efficiency ratio indicated that PET/MR imaging is economical at cost-benefit rates. Cost-effectiveness analyses confirmed positive results for precision and sensitivity values above 76% and 53% at USD 50,000/QALY, and 58% and 44% at USD 100,000/QALY, respectively. The results of these studies suggest that 68 Ga-DOTATATE PET/MR imaging is a cost-effective adjunct for planning postoperative radiation therapy in meningioma patients and that the results can be clinically relevant in terms of sensitivity and specificity.

In [32], the impact of 68Ga-DOTATATE PET imaging on treatment planning for 12 patients with meningiomas who had undergone surgical resection was evaluated. This series comprised two WHO grade I and 10 WHO grade II patients; among the whole group, eight were admitted for recurrent meningiomas and four were newly diagnosed with the disease. Initial treatment planning was based on MRI, and DOTATATE PET imaging was used to refine tumor identification. The findings revealed that 68Ga-DOTATATE PET modified treatment plans in 5 of 12 patients, identifying additional disease foci not detected on MRI in 9 of 12 cases. This modality was particularly useful in detecting intraosseous meningiomas, differentiating tumor recurrence from postoperative changes, and identifying subcentimeter lesions. In conclusion, these results demonstrate the relevance of 68Ga-DOTATATE PET in improving postoperative treatment planning accuracy for meningiomas, especially in challenging cases, and encourage the future application of this method in clinical practice.

Shannon et al. clarified the role of SSTR PET imaging in classifying incidental CNS lesions, often presumed to be meningiomas, based on current clinical practices [33]. A total of 48 patients underwent Ga-68-DOTATATE PET and brain MRI, with incidental CNS lesions identified and predicted to be meningiomas by either imaging modality. The study found that cases with concordant predictions between PET and MRI displayed significantly higher SUV max values (median 7.9 vs. 4.0; *p* = 0.008) and Krenning scores (median 3.0 vs. 2.0; *p* = 0.005) compared to discordant cases. In contrast, lesions with lower SUV max values were more likely to show discordant predictions between Ga-68-DOTATATE PET and MRI. Greater avidity for Ga-68-DOTATATE PET corresponds to certainty in meningiomas, whereas in cases with lower avidity, there was a discrepancy in prediction between imaging modalities.

The Joint EANM/EANO/RANO/SNMMI report provides guidelines for diagnosing and treating meningiomas using radiolabeled SSTR ligands [12], depicting procedural standards for PET with SSTR ligands for meningiomas detection and management. SSTR-ligand PET offers high sensitivity and specificity in detecting meningioma tissue. This provides clinically relevant information that overcomes structural imaging such as MRI or CT scans. The analysis concludes that these guidelines will establish procedure standards for PET imaging in routine practice and clinical trials, facilitating data acquisition, harmonizing interpretations across centers, and facilitating the comparability of studies for the construction of larger databases.

In a case report, a 70-year-old male patient underwent a gallium-68 (68Ga)-DOTATOC brain PET/CT scan to assess a meningeal lesion; the patient was submitted to a previous left foot amputation due to synovial sarcoma and had completed adjuvant chemotherapy three months before the diagnosis. A 68Ga-DOTATOC brain PET/CT was conducted with suspicion of meningioma due to a dural mass. However, the scan showed lower uptake (SUVmax 4.9) than the pituitary gland (SUVmax 9.3), enabling a successful differential diagnosis of metastasis from preexisting malignancies rather than meningioma. Therefore, surgery was proposed; the pathological examination confirmed the diagnosis of dural metastasis of synovial sarcoma. As a matter of fact, meningiomas and metastases are easily distinguished using 68Ga-DOTATOC PET/CT, particularly in patients with a history of malignancy and lesions showing mild uptake [19].

The malignant transformation of meningiomas is a rare condition. Morcet-Delattre et al. [18] described a case of meningioma evolving from WHO grade II atypical to grade III anaplastic metastatic, pathologically proven. In this case report, authors show how PET-CT, using both 68Ga-DOTATOC and 18F-FDG, can be applied to predict tumor grade and stage, and how these imaging techniques show high sensitivity for tumor grading and monitoring.

Milosevic et al. [34] evaluated the correlation between apparent diffusion coefficients (ADCs) and somatostatin receptors (SSTRs) in patients with skull base and orbital space meningiomas. A total of 60 patients with suspected or diagnosed meningiomas underwent 68Ga-DOTATOC PET/MR imaging. ROIs have been traced by extracting ADC values (mean and minimum) and uptake assessed by maximum (SUV max) and mean (SUV mean) in targeted lesions. Pearson correlation coefficients were used to evaluate correlations between these parameters. It was determined that one patient had lymphoma and was excluded from further investigation. As a result, no significant correlations were found between SUV max and ADC min or SUV max and ADC mean. A weak, inverse, but insignificant correlation was found between ADC mean and SUV mean. The study concluded that increased SSTR expression in meningiomas did not correlate with cellularity. SSTR-PET and DWI may offer complementary information on meningioma tumor characteristics.

In a retrospective study by Iglseder and coauthors [25], a correlation analysis between somatostatin receptor subtypes (SSTR 1–5) and the maximum standardized uptake value (SUVmax) in meningioma patients was performed using gallium-68 DOTA-D-Phe1-Tyr3-octreotide positron emission tomography ([68Ga]Ga-DOTATOC) imaging. SUVmax was predicted by a radiomic model based on apparent diffusion coefficient (ADC) maps from diffusion-weighted magnetic resonance imaging (DWI MRI). A total of 51 patients underwent both MRI and [68Ga]Ga-DOTATOC PET before surgery. The ADC maps contained 1940 radiomic features. Using repeated nested cross-validation, a random forest regression model was trained to predict SUVmax. SUVmax values were then compared with SSTR subtype expression in 18 surgical specimens. The random forest regression model successfully predicted SUVmax, with significant a correlation observed in all 100 repetitions (*p* < 0.05). In patients affected by meningiomas with SSTR subtypes 2A/2B, a significant correlation with SUVmax values and the developed radiomic model based on ADC maps has been demonstrated, effectively predicting SUVmax.

Another study by Tatiana et al. [35] explored the use of 68Ga-DOTATOC PET for optic nerve sheath meningioma (ONSM), demonstrating it as a noninvasive, highly sensitive, and specific diagnostic tool. 68Ga-DOTATOC PET was performed on 12 patients with suspected ONSM. In six cases, PET scans confirmed positive uptake (SUVmax > 5), leading to ONSM diagnoses and subsequent radiation therapy for patients experiencing vision loss. In the other six cases, PET scans were negative (SUVmax 5). This included diagnoses such as neurosarcoidosis, cavernous malformation, and uncertain cases that required further investigation. In this study, 68Ga-DOTATOC PET was particularly useful for tumor volume delineation before radiation therapy, reducing unnecessary radiation exposure. This emphasizes the importance of using PET-CT in cases with uncertain diagnoses, especially when MRI alone cannot provide conclusive results. This study concluded that 68Ga-DOTATOC PET enhances the diagnostic approach for intraorbital lesions and plays a significant role in guiding therapeutic management. The diagnosis of these lesions appears to be improved, since an SUVmax ≥ 5 could represent an effective threshold to discriminate an optic nerve sheath meningioma more specifically, avoiding the need for highly invasive biopsy samples. Furthermore, the absorption of 68Ga-DOTATOC, combined with the absence of FDG absorption, has changed and improved the diagnosis of this lesion. Therapeutic management also benefits from the use of this approach, since it is useful for volumetric evaluation of tumors during radiotherapy.

Lütgendorf-Caucig et al. evaluated the potential impact of SSTR PET/CT in monitoring low-grade meningiomas after fractionated proton beam therapy (PBT) compared to standard MRI assessments [36]. A total of 22 patients with WHO grade I meningiomas underwent MRIs and PET/CT scans: 86.4% of patients showed a decrease in SUVmax on PET/CT at the first follow-up; and 81.8% had lower SUVmax, SUVmean, and total lesion activity (TLA) at the last follow-up. MRI showed a 9.3% reduction in tumor volume. In 75% of cases, the heterogeneity index (HI) increased. After radiation, SSTR tracer binding decreased in PET/CT, while tumor volume remained stable or decreased in MRI. This study opens a perspective on researching how radiation therapy changes SSTR PET quantifiers.

The diagnostic advantage of PET imaging in view of the biodistribution of tracer in the entire body permits the evaluation, in the same day, of potential distant malignancy from the primary region. Ghomari et al. reported on a 66-year-old woman with falcine meningioma who had previously received surgery and two radiation treatments for nine years and developed incidental liver lesions three months after the last radiotherapy [37]. Due to the possibility of meningioma metastasis, 68Ga-DOTATOC PET/MRI of the liver and brain, along with a total body PET/CT, revealed hyper-expression of somatostatin receptor 2 in multiple liver and bone lesions. A biopsy of the liver and iliac bone confirmed the metastatic spread of meningioma.

In another study, Aleksandar et al. [38] evaluated [68Ga]-DOTATOC PET/MRI for diagnosing meningiomas. A total of 60 patients with suspected or diagnosed skull base and eye socket meningiomas were included in the study. As a reference standard, histopathology and follow-up imaging were used to compare PET/MRI diagnostic performance with conventional MRI. A total of 60 target lesions were identified, with 54 confirmed as meningiomas. PET/MRI had 95% sensitivity and 75% specificity compared to 96% and 66% for MRI alone. Neither diagnostic accuracy nor local infiltration were significantly different between the two imaging modalities. The accuracy of MRI and PET/MRI in detecting meningiomas was similar, and sequential low-dose SSTR PET/CT might be useful in planning radioligand therapy.

### 3.2. Peptide Radionuclide Receptor Therapy (PRRT) as a Therapeutic Approach to Meningioma

PRRT represents an exciting frontier in therapeutic approaches for meningioma reduction/ablation. Severi et al. applied PRRT in a group of patients affected by recurrent meningiomas following standard surgical and radiotherapy treatments [39]. A set of 42 patients with radiological recurrence of meningiomas were treated with 90Y-DOTATOC (1.1 or 5.5 GBq) or 177Lu-DOTATATE (3.7 or 5.5 GBq) in a mean of four cycles. PET/CT scans with 68Ga-DOTATOC or 111In-octreotide confirmed robust somatostatin receptor uptake. Five patients received 90Y-DOTATOC with a cumulative activity of 11.1 GBq, while 37 patients received 177Lu-DOTATATE with a cumulative activity of 22 GBq. The disease control rate was 57%, with a median progression-free survival of 16 months and a mean overall survival of 36 months. Six patients were re-treated with 177Lu-PRRT, with an average administered activity of 13 GBq over five cycles. The median progression-free survival was 6.5 months, and the average overall survival was 17 months. One patient discontinued treatment due to grade III platelet toxicity and one experienced transient grade II neutropenia.

There have been reports of four patients with recurrent meningiomas of various grades who underwent pretreatment PET/CT with 68Ga-DOTATOC followed by screening for vectorized internal radiation therapy with 177Lu-DOTATATE or before external radiation therapy to facilitate contouring [40]. It was found that in some clinical conditions and without a validated systemic treatment, 177Lu-DOTATATE does not allow reliable contouring. Therefore, 177Lu-PSMA could be a potential adjunct to vectorized internal radiation therapy in these cases. The collected data, analyzed in 2024, showed that in all indications of PET/CT, the detection rate was higher in staging, identification of peptide receptor radioligand therapy (PRRT), and estimation of therapy response. Furthermore, when supported by radiology data, PET was more positive.

PRRT seems promising in different settings of advanced disease after primary treatment. Puranik and coauthors analyzed the role of Lu177-labeled PRRT in eight patients with progressive recurrent high-grade meningiomas who had undergone surgery and/or RT at least once [41]. They received 2.64 MBq/kg of Gallium-68-DOTANOC for PET/CT staging. Acquisitions were made approximately 50 min after intravenous injection. Each patient received at least two cycles of PRRT, with disease response assessed using RANO criteria on MRI and visual analysis of Ga-68 DOTANOC PET/CT uptake. The median time to progression was 8.9 months. Seven patients achieved stable disease after two cycles, while one experienced disease progression. The dosimetric analysis demonstrated high dose delivery and retention using an intra-arterial approach. PRRT was well-tolerated, with no significant peri-procedural or treatment-associated toxicity. These findings suggest that PRRT is a safe and effective therapeutic option for relapsed or refractory meningiomas, with the intra-arterial approach offering improved dose delivery and potential benefits for routine practice.

Furthermore, as second- or third-line treatment of meningioma, another study adopting a PRRT protocol including a four-cycle intravenous regimen of 2–3 MBq/kg of Lutathera^®^ was tested in patients previously treated with surgery, radiosurgery, radiotherapy, and in treatment failures. 68Ga-DOTATOC PET/CT was performed during all monitoring and pre- and post-therapy. The PRRT protocol adopted in this study showed a strong reduction in aggressive multirecurrent meningiomas of WHO grade II. Antitumor activity can be delayed and maintained for 12–18 months after treatment starts. However, a few classes of meningiomas, such as anaplastic meningiomas, did not provide benefit [42].

Similarly, Sylvia et al. [43] conducted the phase II clinical study (NCT03971461) to use 177Lu-DOTATATE, a radiopharmaceutical targeting SSTR2, for treating patients with progressive meningiomas who had not responded to surgery or radiotherapy. A total of 14 patients with intracranial meningiomas were enrolled and received 7.4 GBq (200 mCi) of 177Lu-DOTATATE every eight weeks for four cycles. The primary endpoint, progression-free survival (PFS) at 6 months (PFS-6), was met in 50% of patients. The secondary endpoints, including safety, tolerability, overall survival, and radiographic response, showed that treatment was well tolerated, with stable disease observed in patients who achieved PFS-6. PET imaging with 68Ga-DOTATATE also demonstrated a reduction in tumor uptake in the following cases, indicating potential therapeutic efficacy. The study concluded that 177Lu-DOTATATE treatment was feasible and well-tolerated. Additionally, their findings suggest that 68Ga-DOTATATE PET may be used as a valuable biomarker for assessing therapeutic outcomes in meningiomas.

Another important issue in this setting is the dosimetry assessment. Boursier et al. aimed to improve tumor dosimetry for refractory meningiomas treated with somatostatin receptor-targeted peptide receptor radiotherapy (SSTR-targeted PRRT) [44]. The authors proposed a semi-automated segmentation method on 68Ga-DOTATOC PET images to evaluate the predictive value of SUVmean-derived metrics for tumor-received doses. In total, 39 meningioma lesions from 20 patients were analyzed. Manual segmentation performed by five experienced nuclear medicine medical doctors was used to define PET and SPECT volumes. Tumor-absorbed doses were calculated to adjust for partial volume effects. The optimal semi-automated segmentation method based on a 1.7-fold SUV peak of the meninges had high reproducibility. SUVmean and total lesion uptake (SUVmean × lesion volume) demonstrated better correlations with tumor-absorbed doses than SUVmax, with specific Pearson correlation coefficients ranging from 0.78 to 0.56. An accurate definition of pre-treatment PET volumes is essential, as SUVmean-derived values are the most reliable predictors of tumor-absorbed doses in patients with refractory meningioma treated with 177Lu-DOTATATE. Table 1 shows an overview of the studies on imaging and somatostatin receptor-targeted therapy in meningiomas and neuroaxial tumors discussed in the text. Diagnostic Utility of SSTR PET Imaging (1.1), PRRT Therapy Outcomes (1.2), Imaging Modality Comparisons (1.3), Radiomics and Biomarker Correlations (1.4) and Case Reports and Unique Scenarios (1.5) are considered.

## 4. Conclusions

The advent of somatostatin receptor (SSTR)-targeted radioligands has revolutionized the diagnosis and management of meningiomas, particularly tumors refractory to conventional therapies. 68Ga-DOTA-conjugated PET ligands (e.g., DOTATATE, DOTATOC, DOTANOC) demonstrate superior diagnostic accuracy compared to MRI, especially in detecting occult or recurrent disease. For example, 68Ga-DOTATATE PET identified intraosseous or recurrent lesions in 75% of cases where MRI failed (9/12 patients), while SUVmax thresholds (>50 for paragangliomas vs. 19 for meningiomas) enabled reliable differentiation of neuroaxis tumors. This specificity is critical for guiding stereotactic radiosurgery, as PET/MRI-derived gross tumor volumes (GTVs) were 12.9% larger than MRI-alone volumes (*p* = 0.008), reducing the risk of geographical misses. Furthermore, PET’s prognostic value is underscored by its association with progression risk: patients with negative 68Ga-DOTATOC PET scans remained recurrence-free, whereas positive scans correlated with a 14% local progression rate (*p* = 0.015) and shorter PFS (*p* = 0.029) [28].

For therapeutic applications, 177Lu-DOTATATE peptide receptor radionuclide therapy (PRRT) shows promise in advanced or recurrent meningiomas. In mixed-grade cohorts, PRRT achieved a 6-month progression-free survival (PFS-6) rate of 50%, with a median PFS of 16 months and overall survival (OS) of 36 months in recurrent cases [38]. However, patient selection remains contingent on robust SSTR expression, as insufficient ligand uptake (e.g., in 4/4 grade II/III recurrent tumors) or low SUVmax ratios (e.g., <5 in optic nerve sheath lesions) exclude candidates from PRRT. Quantitative PET metrics, such as SUVmean × tumor volume, further refine therapeutic planning by predicting absorbed radiation doses (Pearson r = 0.78). These findings highlight the dual role of SSTR ligands in both stratifying patients for targeted therapy and optimizing dosimetry.

Despite these advances, limitations persist. Most PRRT studies are retrospective or small (e.g., n = 8–42 [38,40]), and standardized protocols for dosing (e.g., 7.4 GBq vs. 2.64 MBq/kg) or response assessment remain lacking. Additionally, while radiomic models correlating SSTR2A/B expression with SUVmax (*p* < 0.001) offer potential for noninvasive biomarker development, interobserver variability in PET/MRI interpretation and incidental findings (e.g., vertex-to-thigh imaging detected metastases in 1/17 patients) complicate clinical workflows

The present review provides an overview of developments in the diagnostic management of meningiomas using PET imaging and their treatment with radioligand therapy over the last three years, providing evidence of rapid and encouraging progress in both clinical and research settings. To date, PET/CT-guided therapy protocols with 68Ga-DOTATATE have proven highly promising for detecting residual disease after surgery and have also allowed for more specific and targeted radiotherapy planning. Additionally, treatment-refractory meningiomas are difficult to manage due to their constant growth, despite several treatment options including radiotherapy and surgery, and chemotherapy has also failed in many cases. In particular, according to the analyzed literature, in patients with advanced meningiomas that overexpress somatostatin receptor 2, PRRT has been demonstrated to be quite effective and well-tolerated.

The majority of patients who undergo PRRT experience both acute and adverse effects such as nausea, headaches, and, sometimes, vomiting. Additionally, fewer than 3% of patients suffer from chronic adverse effects such as secondary myelodysplastic syndromes and acute myeloid leukemia. [177Lu]Lu-DOTATATE treatment had no apparent nephrotoxicity in the long term. As a result of co-administration of amino acids, some of these symptoms may occur. Nonetheless, little is known about the effects of radiotracer extravasation on soft tissues. In the latter case, discontinuation of treatment is the only recommendation. Patients with meningiomas treated with PRRT may develop acute post-treatment seizures or symptoms of intracranial hypertension, which require treatment with antiepileptic drugs or corticosteroids. Especially in cases of meningiomas in close proximity to the pituitary gland treated with PRRT, it is strongly recommended to monitor the level of hormones in the blood. The available data do not allow correlations between adverse events such as radionecrosis and the location or volume of the tumor. Consequently, in the first cycle of therapy following the injection of [177Lu]Lu-DOTATATE, dosimetry will include SPECT/CT imaging from one hour after the injection until seven days following the injection [46].

The recent advancements in radioligand therapy applied to other diseases, given the possibility of viewing the therapy distribution dose and potential response by using radiopharmaceuticals compounded with a theranostic approach, might be reliable in meningiomas, adding potential value to the clinical outcomes of these patients and limiting radiation exposure and toxicity.

This review takes into account the classification of central nervous system tumors known to date [47] and, in addition, aims to highlight and focus on the rapid progress of nuclear medicine in the diagnosis and treatment of meningiomas through PRRT. A limitation of these state-of-the-art studies on the use of PET and PRRT in these types of brain tumors is represented by the numerous case reports referring to a single patient analyzed, which effectively cannot be translated into statistical analyses on large series of cases. In addition, the reported data are heterogeneous in this sense, as they come from a variety of sources (different hospitals, different brands of instruments, various image sizes, etc.) and have multiple formats [48]. In this regard, the entire manuscript and, more importantly, the reported table are considered valid tools for gaining a comprehensive overview of the articles presented in the last three years. In the future, these summarized data can contribute to giving an extra push to the advancement of AI techniques to support treatment dose optimization and side effects monitoring. In this sense, artificial intelligence strategies, such as radiomics, often support dose optimization and side effects monitoring. We have therefore analyzed and reviewed the latest findings to assess the benefits and safety of these strategies. In the first case, AI, through deep learning algorithms, can conduct more high-impact analyses of clinical data, supporting nuclear medicine physicians, oncologists, and medical physicists in personalized treatment plans. In the second case, algorithms can improve the patient’s quality of life, reducing PRRT long-term risks, thanks to the identification of early signals of possible complications, combining clinical data and features extracted from images. Furthermore, the impact of carbon ion therapy remains unclear due to the limited literature. However, physicians dealing with meningiomas, mainly neurosurgeons and radiation oncologists, should be aware of the multiple possible applications of advanced nuclear medicine techniques for differential diagnosis and potential treatment options in case of recurrent or atypical intracranial meningiomas. In spite of the limited availability of data, major ongoing/recruitment studies indicate a growing interest in previous PRRT applications, reflecting a natural adherence to the theranostic approach. Accordingly, SSR-PET/CT is a promising non-invasive imaging modality for staging NETs, including meningiomas. Theranostics may become a valuable method for detecting this disease early and preventing its progression in the future.

## Figures and Tables

**Table 1:** Studies on somatostatin receptor-targeted imaging and therapy in meningiomas and neuroaxis tumors.

1.1. Diagnostic Utility of SSTR PET Imaging					
Study Population	Patients (n)	Ligand	Key Findings		Ref.
Grade I (WHO)	17 (18 lesions)	68Ga-DOTATATE	PET/MRI increased GTV volume vs. MRI (*p* = 0.008); improved target delineation for radiosurgery.		[26]
Neuroaxis tumors (head/neck)	52 (70 lesions)	68Ga-DOTATATE PET/CT	Paragangliomas (SUVmax 62) vs. schwannomas (SUVmax 2); meningiomas intermediate (SUVmax 19). High SUVmax (>50) aids paraganglioma diagnosis.		[27]
Grade I (WHO)	46 (49 tumors)	68Ga-DOTA-TATE PET/CT	PET findings linked to progression risk (*p* = 0.015) and lower PFS (*p* = 0.029). Negative PET predicted recurrence-free survival.		[28]
Head and neck tumors	30	SSR–PET/CT vs. MRI	PET/MRI detected additional lesions in 9 cases; vertex-to-thigh imaging identified metastases in 1 of 17 patients, with incidental dindings in 8.		[29]
Mixed grades (WHO)	12	68Ga-DOTATATE	PET detected MRI-negative intraosseous lesions and recurrence in 9/12 patients.		[45]
Grade III (suspected meningioma)	1	68Ga-DOTATOC PET/CT	Differentiated dural metastasis (SUVmax 4.9) from meningioma (SUVmax 9.3 in pituitary gland).		[19]
Optic nerve sheath meningioma	12	68Ga-DOTATOC	SUVmax > 5 confirmed ONSM; SUVmax < 5 ruled out tumor (e.g., neurosarcoidosis).		[35]
1.2. PRRT Therapy Outcomes					
Study Population	Patients (n)	Ligand	Dose/Duration	Key Outcomes	Ref.
Grades I–III (WHO)	14	177Lu-DOTATATE	7.4 GBq × 4 cycles	PFS-6: 50%; stable disease, reduced PET uptake.	[43]
Grades I–III (WHO)	8	177Lu-DOTATATE	2.64 MBq/kg × 2 cycles	Median progression: 8.9 months; 7/8 stable disease.	[41]
Recurrent meningiomas	42	90Y-DOTATOC/177Lu-DOTATATE	Cumulative 11–22 GBq	Median PFS: 16 months; OS: 36 months; retreatment PFS: 6.5 months.	[39]
Grades I–IV (WHO)	20 (39 lesions)	177Lu-DOTATATE	7.4 GBq/cycle	SUVmean × volume predicted absorbed dose (r = 0.78); optimal threshold: 1.7× meninges SUVpeak.	[44]
1.3. Imaging Modality Comparisons					
Study Population	Patients (n)	Ligand	Key Findings		Ref.
Mixed grades (WHO)	48	68Ga-DOTATATE vs. MRI	Concordant PET/MRI lesions had higher SUVmax (7.9 vs. 4.0, *p* = 0.008) and Krenning scores.		[33]
Skull base/orbital meningiomas	60	68Ga-DOTATOC	No SUVmax-ADC correlation (*p* > 0.05). PET/MRI specificity (75%) vs. MRI (66%).		[33,37,38]
1.4. Radiomics and Biomarker Correlations					
Study Population	Patients (n)	Ligand	Key Findings		Ref.
Grades I–II (WHO)	51	68Ga-DOTATOC	SSTR2A/B and SSTR5 correlated with SUVmax (*p* < 0.001). Radiomic model predicted SUVmax (r = 0.42).		[25]
Grade I (WHO)	22	68Ga-DOTATOC PET/CT	81.8% showed reduced SUVmean/TLA post-treatment; increased heterogeneity index in 75%.		[36]
1.5. Case Reports and Unique Scenarios					
Study Population	Patients (n)	Ligand	Key Findings		Ref.
Metastatic meningioma	1	68Ga-DOTATOC PET/MRI	Detected liver/bone metastases; biopsy confirmed meningioma spread.		[37]
Recurrent grades II/III	4	177Lu-DOTATATE/PSMA	Insufficient tracer uptake for PRRT eligibility.		[40]

## Data Availability

No new data were created or analyzed in this study.

## Abbreviations

ADC	Apparent diffusion coefficient
DOTA-NOC	DOTA-1-Nal(3)-Octreotide
DOTA-TATE	DOTA-Tyr3-Octreotate
DOTA-TOC	DOTA-Tyr3-Octreotide
EANM	European Association of Nuclear Medicine
EANO	European Association of Neuro-Oncology
GBq	Gigabecquerel
GTV	Gross tumor volume
HI	Heterogeneity index
MRI	Magnetic resonance imaging
NETs	Neuroendocrine tumors
PET	Positron emission tomography
PFS	Progression-free survival
PRRT	Peptide receptor radionuclide therapy
SNMMI	Society of nuclear medicine and molecular imaging
SPECT	Single-photon emission computed tomography
SSR-PET/CT	Somatostatin receptor positron emission tomography/computed tomography
SSTR	Somatostatin receptor
SUV	Standardized uptake value
TLA	Total lesion activity
WHO	World Health Organization
